# Ru@NiMoS aggregate with boosted electrochemical catalysis for enhanced electrochemiluminescence and lidocaine detection

**DOI:** 10.1002/smo.20240011

**Published:** 2024-09-11

**Authors:** Yongzhuang Lu, Haoran Wang, Qiyao Li, Qian Liu, Xiaoxu Zhang, Yuying Jia, Xiangyu Cai, Zheng Zhao, Yanfu Huan, Ben Zhong Tang

**Affiliations:** ^1^ College of Chemistry Jilin University Changchun Jilin China; ^2^ Clinical Translational Research Center of Aggregation‐Induced Emission The Second Affiliated Hospital School of Science and Engineering Shenzhen Institute of Aggregate Science and Technology The Chinese University of Hong Kong Shenzhen Guangdong China; ^3^ Hong Kong Branch of Chinese National Engineering Research Center for Tissue Restoration and Reconstruction and Department of Chemistry The Hong Kong University of Science and Technology Kowloon Hong Kong China; ^4^ Department of Urology Tianjin First Central Hospital Tianjin China

**Keywords:** binder‐free, catalysis, electrochemiluminescence, oxygen evolution reaction, synergistic effect

## Abstract

A binder‐free Ru@NiMoS electrode was engineered by in situ growth of two‐dimensional NiMoS nanosheets on nickel foam. This process effectively promoted the electrostatic‐driven aggregation of Ru(bpy)_3_^2+^, harnessing the synergistic effect to enhance electrochemiluminescence (ECL) performance. The integration (Ru@NiMoS) achieved an impressive ECL efficiency of 70.1%, marking an impressive 36.9‐fold enhancement over conventional Ru. Additionally, its ECL intensity was found to be remarkably 172.2 times greater than that of Ru. Within the Ru(bpy)_3_^2+^/TPA system, NiMoS emerged as a pivotal electrochemical catalyst, markedly boosting both the oxygen evolution reaction and the generation of reactive intermediates. Leveraging these distinctive properties, a highly efficient ECL sensor for lidocaine detection was developed. This sensor exhibited a linear response within the concentration range of 1 nM to 1 μM and achieved a remarkably low detection limit of 0.22 nM, underlining its substantial potential for practical application.

## Graphical abstract

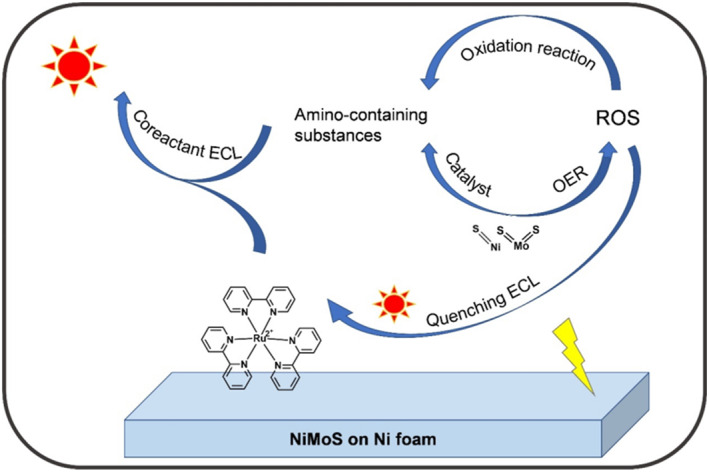

## 1 INTRODUCTION

Electrochemiluminescence (ECL), a luminous phenomenon induced by electron transfer reactions on the electrode surface, was first elucidated in the seminal works of Hercules and Visco in 1964.[^1^, ^2^, ^3^, ^4^] ECL has since gained recognition for its exceptional attributes, including minimal background interference, superior signal‐to‐noise ratios, and precise temporal and spatial control. Over time, the application of various ECL methodologies has proliferated across a wide range of fields, encompassing drug analysis, environmental monitoring, and clinical diagnostics.^[5]^ A notable milestone in the evolution of ECL was the introduction of the Ru(bpy)_3_^2+^ luminophore by Bard and Tokel in 1972, typically utilized in conjunction with tripropylamine (TPA) as a coreactant to form a classic ECL system.^[6]^ However, limitations arise in this configuration due to the suboptimal generation rate of reaction intermediates, impeding significant signal amplification.^[7]^

In the pursuit of fascinating ECL technique, the selection of electrode materials with high electrochemical activity is critical.^[8]^ Recent studies have highlighted the effectiveness of incorporating highly active promoters to refine ECL.^[9]^ The integration of various nanomaterials has been explored, leveraging their extensive surface area, high reaction activity, and favorable biocompatibility to augment sensing device performance.^[10]^ A notable shift in this field is the increasing preference for transition‐metal nanomaterials, particularly over precious metal catalysts, due to their ready availability and promising electrochemical capabilities. Transition metal dichalcogenides (TMDs), characterized by van der Waals interlayer bonding, have emerged as a prominent category of nanomaterials in this regard.^[11]^ Their versatile applications, ranging from photoelectrocatalysis[^12^, ^13^] to energy storage[^14^, ^15^, ^16^] and biomedicine,^[17]^ illustrate their potential for enhancing ECL. Selecting apt nanomaterials is pivotal in the construction of effective ECL devices.^[18]^ Recent advancements have spotlighted the role of MoS_2_‐decorated electrodes as potent signal amplifiers. For instance, the Zhang group's fabrication of a 3D flower‐like MoS_2_ nanomaterials as signal‐promoter in a PTC‐PEI/S_2_O_8_^2−^ system yielded a sensitive detection of methotrexate.^[19]^ Similarly, Chu group investigated the ECL of Ru(bpy)_3_^2+^ at MoS_2_ nanosheets modified electrodes, highlighting the enhancing anodic ECL.^[20]^ Additionally, although NiS has not garnered as much recognition as MoS_2_, it exhibits promising potential in the electronic and optoelectronic realms, suggesting an area ripe for its development in ECL technology.^[21]^

While TMDs like MoS_2_ are recognized as effective coreaction accelerators in ECL systems, challenges persist in the development of TMDs‐based ECL devices. These include limited ECL intensity and efficiency in aqueous environments, as well as the necessity for more efficient methods to integrate the properties of luminophores with the advantageous TMDs. Additionally, most TMDs‐related electrodes are primarily effective in catalyzing coreactions like H_2_O_2_ or glucose, somewhat restricting their broader application scope.^[22]^ Moreover, the impact of concurrent oxygen evolution reaction (OER) processes and the involvement of reactive oxygen species (ROS) in ECL systems are often overlooked and warrant further investigation.^[23]^ Furthermore, a comprehensive mechanism of the multifunctional TMDs in ECL systems hasn't been transparently derived and explicitly explained to the best of our knowledge.

To address the challenges identified earlier, a three‐dimensional faveolate structure composed of MoS_2_ and NiS nanohybrid material (NiMoS) was synthesized and decorated with Ru(bpy)_3_^2+^ through electrostatic attraction to form the Ru@NiMoS electrode with a synergistic effect. The catalytic attributes of NiMoS significantly bolstered the interaction between Ru(bpy)₃^2^⁺ and TPA in the anode ECL reaction, primarily by promoting the OER rate. Consequently, the Ru@NiMoS/TPA system achieved a qualitative leap in signal enhancement (172.2‐fold compared with Ru) and superior ECL efficiency (36.9‐fold compared with Ru). These improvements significantly elevated the analytical performance of immunosensors. Furthermore, the Ru@NiMoS‐based sensor was effectively applied for lidocaine detection, demonstrating a detection limit as low as 0.22 nM.

## 2 RESULTS AND DISCUSSION

**FIGURE 1:**
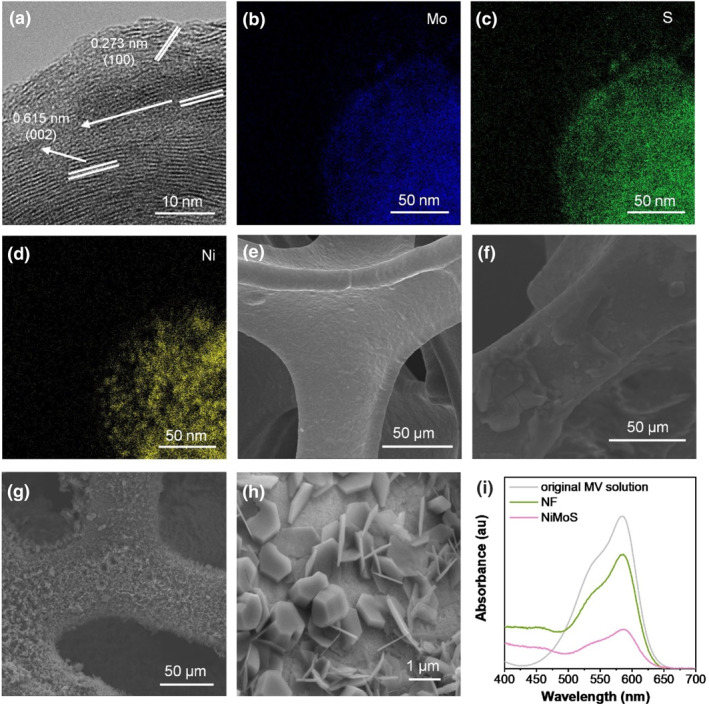
(a) HRTEM image of NiMoS, and corresponding EDS scans for elemental mapping of (b) Mo, (c) S, and (d) Ni. SEM images of (e) NF, (f) Ru, and (g)‐(h) Ru@NiMoS. (i) Dye‐absorption test of NF and NiMoS via UV‐Vis (0.5 × 2 cm^2^ of NF or NiMoS soaked in 10 ml Methyl Violet solution at a concentration of 4 mg L^−1^ and kept in darkness for 18 h).

In this study, NiMoS is crucial to the sensor's performance. In order to investigate the morphological and microstructural characteristics of NiMoS, scanning electron microscopy (SEM) and transmission electron microscopy (TEM) were employed for detailed observation. SEM image (Supporting Information S1: Fig. S1a) revealed that NiMoS displayed a three‐dimensional faveolate structure. The interconnected NiMoS sheets formed irregular pores, providing a substantial accessible surface area. This bidimensional sheet‐like morphology of NiMoS is vividly depicted in Figure 1a. High‐resolution TEM (HRTEM) image exhibited stacked NiMoS with interlayer spacings of 0.615 and 0.273 nm, corresponding to the (002) and (100) planes of MoS_2_, respectively. The selected area electron diffraction patterns (Supporting Information S1: Fig. S1b) confirmed these observations with two sets of concentric rings. Transmission electron microscopy energy‐dispersive X‐ray spectroscopy (EDS) scans of the NiMoS composites (Figure 1b–d) revealed a distinct partitioned distribution of Ni and Mo within the ultrathin nanosheet structure, with NiS exhibiting a point‐like distribution within the NiMoS matrix. These evidences confirmed the successful synthesis of MoS_2_ and NiS hybrid structures.

SEM image in Figure 1e depicts the smooth surface of baer nickel foam (NF). In Figure 1f, the weeny peeled flakes of Ru(bpy)_3_^2+^ adhered to NF were observed with Nafion as a binder. Contrasting, Ru@NiMoS (Figure 1g) exhibited a thick, furry surface, indicative of massive active materials. Enlarged SEM image (Figure 1h) highlighted the regular shape of Ru(bpy)_3_^2+^, emphasizing the efficient absorption via electrostatic attraction. Zeta potential measurements confirmed this electrostatic attraction, with NiMoS exhibiting a potential of −24.12 mV, suggesting the immobilization of positively charged Ru(bpy)_3_^2+^ on the negatively charged NiMoS. The sulfur atoms on the NiMoS nanosheet surface offer vacant orbitals for the efficient immobilization of cationic Ru(bpy)_3_^2+^.[^24^, ^25^] EDS of the as‐prepared Ru@NiMoS composites (Supporting Information S1: Fig. S2a‐g) demonstrated distributions of Ru, Cl, O, N, S, Ni, and Mo elements, indicating effective cross‐linking of NiMoS and Ru(bpy)_3_^2+^ (Figure 1h). Supporting Information S1: Fig. S3 delineated the comprehensive structural characterization of the synthesized composites, ascertained through X‐ray diffraction (XRD) and X‐ray photoelectron spectroscopy (XPS) analyses. X‐ray diffraction revealed distinct diffraction peaks of MoS_2_ (JCPDS 37–1492), NiS (JCPDS 12–0041) and Ru(bpy)_3_^2+^ aligning with extant literature,[^26^, ^27^, ^28^, ^29^, ^30^, ^31^, ^32^, ^33^, ^34^, ^35^, ^36^] and affirmed minimal impact of Ru(bpy)_3_^2+^ incorporation on the NiMoS crystal lattice. X‐ray photoelectron spectroscopy analysis further elucidated the elemental chemical state via the Mo 3 days, Ni 2p and S 2p core level spectra. The analytical outcomes collectively substantiated that the chemical integrity of Ru(bpy)_3_^2+^ is retained in the Ru@NiMoS sample. Notably, Ru@NiMoS exhibited an augmented Mo:S ratio compared to NiMoS in Supporting Information S1: Table S1, indicative of the formation of Mo‐O and Ru‐S bonds. Comparative assessment of the Ni:S ratios highlighted a diminution of sulfur atoms on the Ru@NiMoS surface, suggesting a pivotal role of sulfur in stabilizing ruthenium within the matrix.[^37^, ^38^] The findings from EDS, XRD, and XPS cohesively pointed towards the successful anchoring of Ru(bpy)_3_^2+^ onto NiMoS structure. UV‐visible (UV‐vis) spectroscopy (Figure 1i) compared the absorption of methylene blue in the original and solutions soaked with NF and NiMoS. The characteristic peaks at 520 and 580 nm revealed lower absorbance in the NiMoS‐soaked solution compared to NF, indicating more dye molecules adsorbed on the NiMoS surface. This confirmed the increased accessible surface area of NiMoS, essential for abundant active catalytic sites.

**FIGURE 2:**
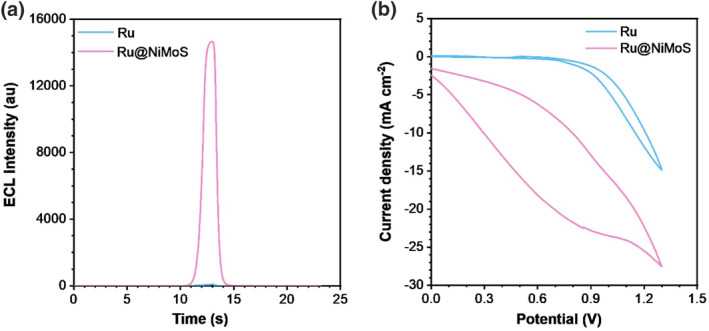
(a) ECL profiles and (b) corresponding CV curves for Ru and Ru@NiMoS (Photomultiplier Tube (PMT): 500 V, electrolyte: 0.075 M PBS (pH 11) with 20 mM TPA, potential range: 0–1.3 V, scan rate: 120 mV s^−1^).

To optimize the ECL enrichment of Ru@NiMoS, several parameters were methodically adjusted, including scan rate, phosphate buffered saline (PBS) concentration, TPA concentration, and the pH level of the electrolyte, as elaborated in Supporting Information S1: Fig. S4. The most conducive conditions were identified as 0.075 M PBS with 0.02 M TPA as the electrolyte at a pH of 11, and a scan rate of 120 mV s^−1^. Comparative ECL experiments of Ru and Ru@NiMoS were conducted under these optimized conditions in Figure 2a. Ru exhibited a faint signal at 97 au, attributed to inherent auto‐luminescence of Ru(bpy)_3_^2+^. Whereas the extraordinary luminescence of Ru@NiMoS reached a maximum intensity of 16,708 au, dramatically surpassing about 172.2‐fold compared to Ru. To elucidate how the NiMoS substrate influences ECL, the luminescent property of NiMoS alone was investigated in Supporting Information S1: Fig. S5. NiMoS did not generate any luminescent response at its current peak position, thereby confirming its intrinsic non‐luminescent characteristics. Further analysis through cyclic voltammetry, as presented in Figure 2b, identified apparent current shoulders at 1.3 V for both samples, which aligned with the OER behaviors within the constrained potential window of aqueous solutions, theoretically at 1.229 V.^[39]^ Notably, Ru@NiMoS displayed a significantly enhanced electro‐active nature compared to Ru, indicative of the superior oxidoreduction capacity, which is attributed to the MoS_2_ component served as a reservoir for electron redistribution.^[40]^ Moreover, the anodic current peak of Ru@NiMoS was 13.2 times higher than that of Ru at 0.8 V, consistent with the generation of free radical cations of TPA.[^41^, ^42^, ^43^] Besides, the ECL efficiency of Ru and Ru@NiMoS was calculated based on the relative ratio of integrated photon emission to electron transport rate. Ru@NiMoS achieved a remarkable ECL efficiency of 70.1%, which is 36.9 times higher than that of Ru. Repeatability also occupies critical importance. As depicted in Supporting Information S1: Fig. S5, Ru@NiMoS exhibited consistent performance across 10 separate scans, with a relative standard deviation of only 0.53%, highlighting its reliability.

**FIGURE 3:**
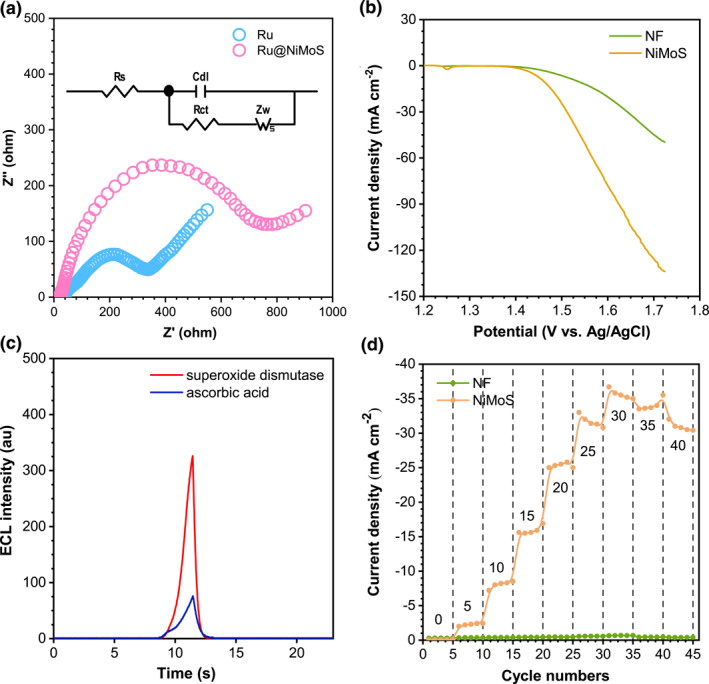
(a) EIS for Ru and Ru@NiMoS recorded at open circuit voltage, the inset is typical equivalent circuit (frequency range: 0.01 Hz ‐ 1000 kHz, amplitude: 5 mV, electrolyte: 0.1 M KCl and 5 mM [K_3_Fe(CN)_6_/K_2_Fe(CN)_6_]). (b) iR‐corrected LSV curves for NF and NiMoS (sweep scan rate: 5 mV s^−1^, electrolyte: 1 M KOH). (c) ECL spectra of Ru@NiMoS with 0.1 mM superoxide dismutase as the O_2_*^‐^ scavenger or 0.1 mM ascorbic acid as the O_2_*^‐^, OH* and HO_2_*^‐^ scavenger (PMT: 500 V, electrolyte: 0.075 M PBS (pH 11) with 20 mM TPA, potential range: 0–1.3 V, scan rate: 120 mV s^−1^). (d) Effects of various TPA concentrations on the current responses of NF and NiMoS in 0.075 M PBS (TPA amounts: 0, 5, 10, 15, 20, 25, 30, 35, and 40 mM).

Electrochemical Impedance Spectroscopy was employed to assess the correlation between the electrochemical properties and the ECL effects of Ru and Ru@NiMoS, as depicted in Figure 3a. An equivalent circuit (shown in the inset) determined the faradaic interfacial charge transfer resistance (*R*_*ct*_) at high frequency, and the *R*_*ct*_ values were found to be 374 *Ω* for Ru and 748 *Ω* for Ru@NiMoS. This increased conductivity for Ru@NiMoS was attributed to the agglomeration of a substantial amount of NiMoS and Ru(bpy)_3_^2+^. Therefore, the altered conductivity of the ruthenium complex electrode was not the reason for promoting the ECL signal, but posed limitations on electrode kinetics and the efficiency of electrochemical reactions.

The OER activities were investigated employing linear sweep voltammetry (LSV) curves at a scan rate of 5 mV s^−1^ in an alkaline environment, as delineated in Figure 3b. Both Ru and NiMoS exhibited an onset potential of 1.4 V, and the current intensity of NiMoS escalated markedly compared to Ru, culminating in the final current density of NiMoS being thrice that of Ru. A critical aspect of the OER process involved the generation of singlet oxygen (O_2_), which was capable of being adsorbed by the active sites on NiMoS. Subsequently, O_2_ was catalyzed to produce ROS, such as superoxide radicals (O_2_*‐), hydroxyl radicals (OH*), and hydrogen superoxide free radicals (HO_2_*‐).^[44]^ According to references, these ROS are instrumental in augmenting the ECL response.^[45]^ Moreover, the porous structure of NiMoS offered channels for oxygen to rapidly diffuse to the active sites, prolonging its interaction with the NiMoS catalyst, thereby fostering ROS generation. Figure 3c illustrated the ECL behavior of Ru@NiMoS in the presence of radical scavengers, definitively confirming the involvement of ROS in the Ru(bpy)_3_^2+^/TPA system. The impact of O_2_*‐ was examined using superoxide dismutase that eliminated O_2_*^−^. This intervention led to a sharply decrease in the ECL signal, as distinctly illustrated in Figure 3c (red line). Further investigations into the formation and influence of other oxygen‐derived intermediates in the ECL process were pursued by studying the ECL response following the removal of HO_2_*^‐^, OH*, and O_2_*^‐^ in the presence of ascorbic acid. As shown in Figure 3c (blue line), continuously decreased ECL intensity was observed, reinforcing the pivotal role of the ROS pathway in the ECL reaction. Figure 3d displayed the influence of various TPA concentrations (0–40 mM) on the current responses of NF and NiMoS, aimed at assessing the catalytic efficiency of NiMoS_2_ as a coreaction promoter. The current density of NF showed marginal increase with escalating TPA concentrations, whereas the current density of NiMoS displayed a significantly enhancement, underscoring superior electrocatalytic activity of NiMoS in facilitating the decomposition of TPA. Above all, these findings not only corroborated the crucial role of ROS but also affirmed the catalytic facilitation provided by NiMoS in the coreaction ECL process.

**FIGURE 4:**
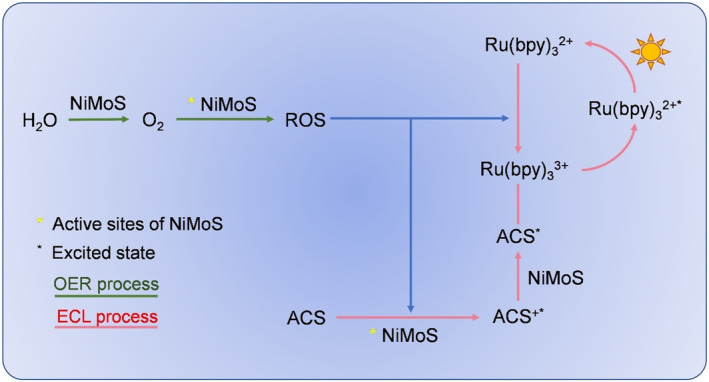
ECL mechanism of the Ru@NiMoS/ACS coreactant system.

As amino functional groups play a crucial role as coreaction in the ECL reaction with the luminophore Ru(bpy)_3_^2+^.[^46^, ^47^, ^48^, ^49^] A comprehensive mechanistic contributions of multifunctional NiMoS within the ruthenium complexes‐based coreactant system was delineated in Figure 4. Initially, NiMoS demonstrated remarkable OER efficiency, leading to the generation of substantial amounts of O_2_ (Equation 1). O_2_ was subsequently adsorbed and catalytically processed at the oxygen vacancy sites of NiMoS nanosheets, resulting in the production of ROS (Equation 2). These ROS, characterized by their potent oxidizing capabilities, efficiently oxidized Ru(bpy)_3_^2+^ and amine‐containing substances (ACS). The cooperation of ROS and the active sites of NiMoS markedly expedited the transmutation of coreactant and luminophore into exorbitant radical ions (Equations 3 and 4). The generated ACS*^+^ cation involved deprotonation from the *α*‐carbon to form ACS*, a process that was modulated by the proton‐absorbing capacity of the alkaline supporting electrolyte (Equation 5). This step was pivotal as it is rate‐limiting, given the short half‐life of the intermediates (*t*_1/2_–200 μs).^[50]^ Furthermore, the substantial electron transfer from ACS × to the π* orbital of the Ru(bpy)_3_^3+^ ligand facilitated the excitation of Ru(bpy)_3_^2+^* (Equation 6). Eventually, the excited Ru(bpy)_3_^2+^* species decayed to the ground state upon scanning to 1.3 V, releasing energy as a pronounced optical signal (Equation 7). Overall, NiMoS functioned as the bridging species for ECL and OER reactions with the generated ROS. The nano‐NiMoS material not only efficiently enriched Ru(bpy)_3_^2+^, but also exhibited catalytic activity towards the coreactant, thereby increasing the localized concentration of active excited‐state ions on the electrode surface, which in turn leads to the enhanced ECL performance.

(1) $$H_{2}O+e^{-}\overset{\text{NiMoS}}{\to }O_{2}$$

(2) $$O_{2}+e^{-}\overset{\text{NiMoS}}{\to }\text{ROS}$$

(3) $${\text{Ru}\left(\text{bpy}\right)}_{3}^{2+}+\text{ROS}+2H^{+}+3e^{-}\overset{\text{NiMoS}}{\to }{\text{Ru}\left(\text{bpy}\right)}_{3}^{3+}+{\text{OH}}^{-}$$

(4) $$\text{ACS}+\text{ROS}+2H^{+}+2e^{-}\overset{\text{NiMoS}}{\to }{\text{ACS}}^{*+}+{\text{OH}}^{-}$$

(5) $${\text{ACS}}^{*+}\overset{\text{NiMoS}}{\to }{\text{ACS}}^{*}+H^{+}$$

(6) $${\text{Ru}\left(\text{bpy}\right)}_{3}^{3+}+{\text{ACS}}^{*}\to {\text{Ru}\left(\text{bpy}\right)}_{3}^{*2+}+\text{product}$$

(7) $${\text{Ru}\left(\text{bpy}\right)}_{3}^{*2+}\to {\text{Ru}\left(\text{bpy}\right)}_{3}^{2+}+\text{hv}$$

**FIGURE 5:**
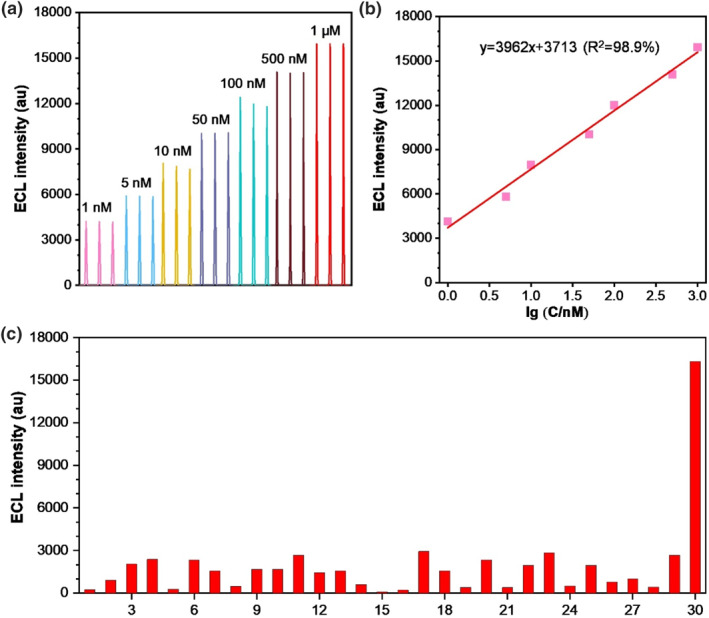
Lidocaine detection based on the Ru@NiMoS sensor: (a) ECL intensity plotted against various concentrations of lidocaine (1 nM, 5 nM, 10 nM, 50 nM, 100 nM, 500 nM, and 1 μM); (b) Linear correlation between ECL intensity and the logarithm of lidocaine concentration; (c) Sensitivity tests with 1 μM lidocaine or other amine‐containing substances. (PMT: 800 V, electrolyte: 0.075 M PBS (pH 11) with 100 μM NO_2_^−^, potential range: 0–1.3 V, scan rate: 120 mV s^−1^. Serial numbers 1–30 represent glycine, creatinine, adenine, hydroxylamine hydrochloride, cytosine, *L*‐Phenylalanine, thioacetamide, vitamin E, ibuprofen, carboprost, vitamin A, vitamin D, vitamin B1, vitamin B5, salicylic acid, levobunolol, diclofenac, glimepiride, fluoxetine, adenosine triphosphate, phenylbutazone, leuprorelin, metronidazole, *L*‐Asparagine, adenosine triphosphate, coenzyme A, lactate, and urea, *DL*‐Valine and lidocaine, respectively).

Ru@NiMoS demonstrated remarkable potential as a highly sensitive sensor platform. Lidocaine, an anesthetic and antiarrhythmic drug historically plagued by sensitivity and selectivity challenges, was employed as coreactant to Ru(bpy)_3_^2+^.[^51^, ^52^] In this study, a signal‐on sensor utilizing a Ru@NiMoS electrode was developed to facilitate enhanced ECL analysis of lidocaine, incorporating nitrite (NO_2_^−^) as a specific screening agent.^[53]^ This inclusion of NO_2_^−^ effectively differentiated lidocaine from other ACS, thus elevating the assay specificity. As depicted in Figure 5a, a direct proportional relationship was discerned between the ECL intensity and lidocaine concentration, spanning a broad linear range from 1 nM to 1 μM. Notably, consistent luminescence intensity values were replicated across three independent experiments. Figure 5b further delineated a desirable linear correlation between ECL intensity and the logarithm of lidocaine concentration with the standard fitting equation being *I*_*ECL*_ = 3962lg_(*C*)_+3713 (*R* ^2^ = 0.989). The limit of detection was determined to be an impressively low 0.22 nM. Figure 5c presented the ECL responses in the presence of 29 different potential interferences to evaluate the selectivity. Remarkably, the ECL intensity for these substances did not surpass the detection threshold specific to lidocaine. The feasibility of Ru@NiMoS‐based sensors for analyzing lidocaine in human serum samples was investigated, and as detailed in Supporting Information S1: Table S2, the sensor demonstrated reasonable recovery rates across varying concentrations (10 μM, 1 μM, and 100 nM), ranging from 92.6% to 103.4%, 91%–109%, and 97.23%–106.73%, respectively. These findings validated the superior sensitivity and selectivity of the designed sensor, thereby reinforcing its potential as a highly effective tool for the precise detection of lidocaine.

## 3 CONCLUSION

In summary, this study presented a comprehensive investigation into the integration of NiMoS within the Ru(bpy)_3_^2+^/TPA ECL system, where NiMoS acted both as an electrocatalyst and a structure framework. Owing to its outstanding OER and oxidation catalytic activities, the generation of active free radicals was significantly enhanced, thereby boosting the ECL efficacy. The synergistic effect between NiMoS and Ru(bpy)_3_^2+^ led to exceptional ECL performance, with the ECL intensity of Ru@NiMoS being 172.2 times greater than that of Ru, and the Ru@NiMoS ECL efficiency of 70.1%—an improvement of 36.9 over Ru. Additionally, the amplificatory signal strategy was applied to probe lidocaine, achieving a remarkable low detection limit of 0.22 nM. This strategy provides a promising pathway for the feasible preparation and reference mechanism of the future ECL systems involving TMDs.

## 4 EXPERIMENTAL SECTION

The synthesis of Ru@NiMoS involved a facile two‐step process, encompassing hydrothermal treatment and subsequent incubation in Sch. 1. Initially, a 3 × 2 cm^2^ piece of NF, underwent ultrasonic cleaning in acetone, ethanol, and deionized water for 20 min each. Subsequently, a 20 ml homogeneous solution was prepared by vigorously stirring a mixture of 4 mM Na_2_MoO_4_ and 8 mM thiourea. The pre‐cleaned NF was then immersed in this precursor solution. This assembly was transferred to a teflon‐lined autoclave and maintained at 180°C for 24 h. The resultant product was thoroughly rinsed thrice with deionized water and alcohol, and subsequently dried in a vacuum oven at 80°C for 4 h to yield NiMoS. In the final step, NiMoS was immersed in a 10 mM Ru(bpy)_3_^2+^ solution, facilitating the fixation of Ru(bpy)_3_^2+^ onto the electrode surface through electrostatic attraction, culminating in the formation of Ru@NiMoS. For comparative purposes, Ru was prepared by immersing cleaned NF in a 10 mM Ru(bpy)_3_^2+^ suspension with Nafion as binder, followed by drying at room temperature.

**Scheme 1:**
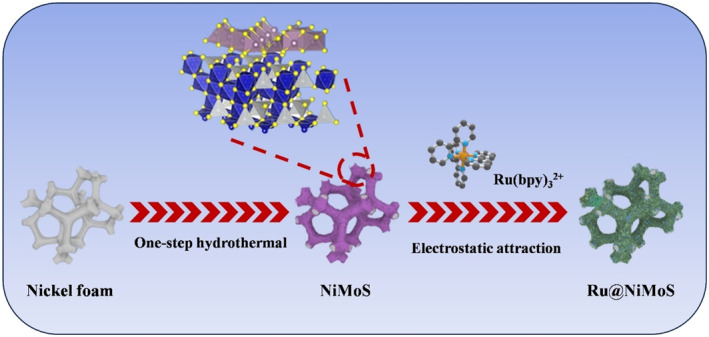
Schematic illustration of the construction process of Ru@NiMoS.

## CONFLICT OF INTEREST STATEMENT

The authors declare no conflicts of interest.

## ETHICS STATEMENT

No animal or human experiments were involved in this study.

## Supporting information

Supporting information S1

## Data Availability

The data that support the findings of this study are available in the supplementary material.

## References

[1] P. Bertoncello , R. J. Forster , Biosens. Bioelectron. 2009, 24, 3191. 19318243 10.1016/j.bios.2009.02.013

[2] B. Yin , L. Jiang , X. Wang , Y. Liu , K. Y. Kuang , M. M. Jing , C. M. Fang , C. J. Zhou , S. Chen , M. Z. Zhu , Aggregate 2023, 5, e417.

[3] D. M. Hercules , Science 1964, 145, 808. 17816303 10.1126/science.145.3634.808

[4] R. E. Visco , E. A. Chandross , J. Am. Chem. Soc. 1964, 86, 5350.

[5] H. Gao , S. Y. Shi , S. M. Wang , Q. Q. Tao , H. L. Ma , J. Hu , H. Y. Chen , J. J. Xu , Aggregate 2023, e394.

[6] L. Zhang , S. Dong , Anal. Chem. 2006, 78, 5119. 16841937 10.1021/ac060451n

[7] Y. Y. Sun , Q. Yan , Z. Jun , Q. X. Ren , Microchim. Acta 2021, 188, 300.

[8] Y. Zhou , E. Villani , T. Kurioka , Y. Zhang , I. Tomita , S. Inagi , Aggregate 2023, 4, e202.

[9] H. Wang , Anal. Bioanal. Chem. 2021, 413, 4119. 33715042 10.1007/s00216-021-03247-1

[10] H. P. Sun , H. Wang , W. Q. Bai , L. Bao , J. X. Lin , Y. Lia , Talanta 2019, 191, 350. 30262070 10.1016/j.talanta.2018.08.070

[11] H. Y. Wang , L. C. Tian , X. Zhao , M. Ali , K. Yin , Z. C. Xing , Mater. Today Commun. 2023, 34, 105041.

[12] S. Pahra , S. Sharma , P. Devi , Green Energy Harvesting: Materials for Hydrogen Generation and Carbon Dioxide Reduction 2022, pp. 46–74.

[13] R. Ghosh , B. Papnai , Y. S. Chen , K. Yadav , R. Sankar , Y. P. Hsieh , Y. F. Chen , Adv. Mater. 2023, 2210746. 10.1002/adma.202210746 36756807

[14] P. Prabhu , V. Jose , J. M. Lee , Matter 2020, 2, 526.

[15] B. Chen , D. Chao , E. Liu , M. Jaroniec , N. Zhao , S. Z. Qiao , Energy Environ. 2020, 13, 1096.

[16] Q. Yun , Q. Lu , X. Zhang , C. Tan , H. Zhang , Angew. Chem. Int. Ed. Engl. 2018, 57, 626. 28834184 10.1002/anie.201706426

[17] X. Zhang , Z. Lai , Q. Ma , H. Zhang , Chem. Soc. Rev. 2018, 47, 3301. 29671441 10.1039/c8cs00094h

[18] W. Xiang , Y. Luo , Y. Yue , H. Ding , Y. Dong , J. Electroanal. Chem. 2022, 915, 116364.

[19] P. J. Wang , Q. Wu , C. X. Wang , Y. X. Pu , M. Zhou , M. X. Zhang , J. Electrochem. Soc. 2020, 167, 107505.

[20] Y. Zhang , H. Yin , C. B. Jia , Y. P. Dong , H. C. Ding , X. F. Chu , Spectrochim. Acta Mol. Biomol. Spectrosc. 2020, 240, 118607. 10.1016/j.saa.2020.118607 32593843

[21] C. Ma , H. F. Wei , M. X. Wang , S. J. Wu , Y. C. Chang , J. R. Zhang , L. P. Jiang , W. L. Zhu , Z. X. Chen , Y. H. Lin , Nano Lett. 2020, 20, 5008. 32515975 10.1021/acs.nanolett.0c01129

[22] L. Shang , X. H. Zhao , W. Zhang , L. P. Jia , R. N. Ma , Q. W. Xue , H. S. Wang , A. X. Guo , L. Si , Microchim. Acta 2022, 189, 17.

[23] Y. Qin , Z. Wang , J. Xu , F. Han , X. Zhao , D. Han , Y. Liu , Z. Kang , L. Niu , Anal. Chem. 2020, 92, 15352. 33170643 10.1021/acs.analchem.0c02568

[24] X. J. Li , X. Sun , D. W. Fan , T. Yan , R. Feng , H. Wang , D. Wu , Q. Wei , Biosens. Bioelectron. 2019, 142, 111551. 31382095 10.1016/j.bios.2019.111551

[25] A. Diallo , A. C. Beye , T. B. Doyle , E. Park , M. Maaza , Green Chem. Lett. Rev. 2015, 8, 30.

[26] J. Marques , L. Anjo , M. P. M. Marques , T. M. Santos , F. A. A. Paz , S. S. Braga , J. Organomet. Chem. 2008, 693, 3021.

[27] J. Zhou , R. Zong , J. Ye , J. Lumin. 2007, 122, 218.

[28] S. Chinnathambi , M. Ammam , J. Power Sources 2015, 284, 524.

[29] T. A. Ho , Y. B. Cho , Y. S. Kim , J. Nanosci. Nanotechno. 2016, 16, 4534. 10.1166/jnn.2016.11008 27483786

[30] L. F. Yang , L. Zhang , G. C. Xu , X. Ma , W. W. Wang , H. J. Song , D. Z. Jia , ACS Sustain. Chem. Eng. 2018, 6, 12961.

[31] M. X. Shang , C. C. Du , H. Huang , J. X. Mao , P. Liu , W. B. Song , J. Colloid Interface Sci. 2018, 532, 24. 30077063 10.1016/j.jcis.2018.07.127

[32] D. Siddhartha , T. Abhishek , H. Shamima , Mater. Today: Proc. 2021, 46, 6127.

[33] H. Y. Nan , Z. L. Wang , W. H. Wang , Z. Liang , Y. Lu , Q. Chen , D. W. He , P. H. Tan , F. Miao , X. R. Wang , J. L. Wang , Z. H. Ni , ACS Nano 2014, 8, 5738. 24836121 10.1021/nn500532f

[34] X. B. Xu , W. Zhong , X. Zhang , J. Dou , Z. G. Xiong , Y. Sun , T. T. Wang , Y. W. Du , J. Colloid Interface Sci. 2019, 543, 147. 30797998 10.1016/j.jcis.2019.02.054

[35] M. A. Bissett , I. A. Kinloch , R. A. Dryfe , ACS Appl. Mater. Interfaces 2015, 7, 17388. 26196223 10.1021/acsami.5b04672

[36] S. Baik , Y. Koo , W. Choi , Curr. Appl. Phys. 2022, 42, 38.

[37] K. Saha , S. Gayen , U. Kaur , T. Roisnel , S. Ghosh , Dalton Trans. 2021, 50, 12990. 34581334 10.1039/d1dt01614h

[38] S. Dam , A. Thakur , S. Hussain , Mat. Sci. Semicon. Proc. 2021, 136, 106162.

[39] W. Sun , L. Cao , J. Yang , J. Mater. Chem. A 2016, 4, 12561.

[40] S. Hao , X. Ji , F. Liu , S. Zhong , K. Y. Pang , K. G. Lim , T. C. Chong , R. Zhao , ACS Appl. Nano Mater. 2021, 4, 1766.

[41] G. Kalaiyarasan , C. V. Raju , M. Veerapandian , S. S. Kumar , J. Joseph , Anal. Bioanal. Chem. 2020, 412, 539. 31838557 10.1007/s00216-019-02305-z

[42] Y. P. Dong , T. T. Gao , Y. Zhou , L. P. Jiang , J. J. Zhu , Sci. Rep. 2015, 5, 15392. 26472243 10.1038/srep15392 PMC4607998

[43] J. X. Guo , H. F. Zhu , Y. F. Sun , L. Tang , X. Zhang , Electrochim. Acta 2016, 211, 603.

[44] X. Tan , W. Ding , Z. Jiang , L. Sun , Y. Huang , Nano Res. 2022, 1.

[45] F. Takahashi , R. Shimizu , T. Nakazawa , J. Y. Jin , Ultrason. Sonochem. 2020, 63, 104947. 31952005 10.1016/j.ultsonch.2019.104947

[46] X. Li , X. Du , Microchem. J. 2022, 176, 107224.

[47] S. J. Wang , S. Zhu , Z. Q. Kang , X. X. Wang , Z. X. Deng , K. Hu , J. J. Hu , X. C. Liu , G. X. Wang , G. C. Zang , Y. C. Zhang , Research 2023, 6, 0117. 37287888 10.34133/research.0117 PMC10243198

[48] Y. Q. Han , Y. F. Fang , X. T. Ding , J. Liu , Z. Y. Jin , Y. H. Xu , Electrochem. Commun. 2020, 116, 106760.

[49] A. Krishnakumar , R. K. Mishra , S. Kadian , A. Zareei , U. H. Rivera , R. Rahimi , Anal. Chim. Acta 2022, 1229, 340332. 36156230 10.1016/j.aca.2022.340332

[50] Y. Wang , J. Ding , P. Zhou , J. Liu , Z. Qiao , K. Yu , J. Jiang , B. Su , Angew. Chem. Int. Ed. Engl. 2023, 62, e202216525. 36812044 10.1002/anie.202216525

[51] Y. Han , Y. Fang , X. Ding , J. Liu , Z. Jin , Y. Xu , Electrochem. Commun. 2020, 116, 106760.

[52] F. Takahashi , K. Matsuda , T. Nakazawa , S. Mori , M. Yoshida , R. Shimizu , H. Tatsumi , J. Jin , Separ. Sci. 2023, 6, 2200081.

[53] Y. Lu , Z. Zhao , X. Zhang , Y. Jia , H. Shan , Y. Huan , B. Z. Tang , Sens. Actuators B Chem. 2023, 396, 134590.

