# Supplementary material for: Ru@NiMoS aggregate with boosted electrochemical catalysis for enhanced electrochemiluminescence and lidocaine detection
Source: Smart Mol. 2024 Sep 11;3(1):e20240011. doi: 10.1002/smo.20240011 (PMC12117923; doi:10.1002/smo.20240011)
Supplement: Supplementary file 1 — Supporting information S1 [file SMO2-3-e20240011-s001.docx]

Supporting Information

Ru@NiMoS Aggregate with Boosted Electrochemical Catalysis for Enhanced Electrochemiluminescence and Lidocaine Detection

Yongzhuang Lu,^1,2^ Haoran Wang,^3^ Qiyao Li,^2^ Qian Liu,^4^ Xiaoxu Zhang,^1^ Yuying Jia,^1^ Xiangyu Cai,^1^ Zheng Zhao,^2*^ Yanfu Huan,^1*^ and Ben Zhong Tang^2,3*^

^1^ *College of Chemistry, Jilin University, Changchun, 130012, Jilin, P. R. China*

^2^ *Clinical Translational Research Center of Aggregation-Induced Emission, The Second Affiliated Hospital, School of Science and Engineering, Shenzhen Institute of Aggregate Science and Technology, The Chinese University of Hong Kong, Shenzhen (CUHK-Shenzhen), Guangdong 518172, China*

^3^ *Hong Kong Branch of Chinese National Engineering Research Center for Tissue Restoration and Reconstruction and Department of Chemistry, The Hong Kong University of Science and Technology, Kowloon, Hong Kong, China*

^4^ *Department of Urology, Tianjin First Central Hospital, Tianjin 300192, China*

*Contact of the corresponding author:* *[zhaozheng@cuhk.edu.cn](mailto:zhaozheng@cuhk.edu.cn) (Z. Zhao), yfhuan@jlu.edu.cn (Y. Huan)* *and* [*tangbenz@cuhk.edu.cn*](mailto:tangbenz@cuhk.edu.cn) *(B. Z. Tang).*

**Content**

**Experiment**

Chemical and reagents,

Characterization apparatus,

Electrochemiluminescence measurements,

Prepared sensor for Lidocaine detection.

**Fig. S1.** SEM and SEAD of NiMoS.

**Fig. S2.** EDS elemental mapping of Ru@NiMoS.

**Fig. S3.** XRD pattern and XPS high resolution spectra of NiMoS, Ru, and Ru@NiMoS.

**Fig. S4.** Optimization of scan rate, PBS concentration, TPA concentration, and pH for Ru@NiMoS.

**Fig. S5.** Repeated ECL tests of Ru@NiMoS.

**Fig. S6.** Synchronized ECL and CV curves of NiMoS.

**Table S1.** Atomic percent derived from XPS data for NiMoS and Ru@NiMoS.

**Reference**

## **Experiment**

## **Chemical and reagents**

All the reagents were of analytical grade and used without further purification. [Ru(bpy)_3_]Cl_2_.6H_2_O, NaNO_2_, NaMoO_4_, TPA, thiourea, glycine, creatinine, adenine, hydroxylamine hydrochloride, cytosine, thioacetamide, adenosine triphosphate, coenzyme A, lactic acid, urea, L- Phenylalanine, L-asparagine, DL-valine, salicylic acid, glimepiride, metronidazole, and lidocaine were purchased from Anaiji Chemical Co., Ltd (Anhui, China). K_2_HPO_4_ was purchased from Guangfu Fine Chemical Research Institute (Tianjin, China). KOH and KCl were bought from Tiantai Chemical Co., Ltd (Tianjin, China). HCl was purchased from Xinguang Chemical Reagent Factory. KH_2_PO_4_ was bought from Beijing Chemical works. K_3_[Fe(CN)_6_] and K_4_[Fe(CN)_6_] were purchased from Beijing Chemical Reagent Research Institute. Urea was bought from Jingjin Chemical Factory (Tianjin). Zobromol was purchased from Xi'an Zhongtian Biopharmaceutical Co., Ltd. Ibuprofen was purchased from Sino-US Tianjin SmithKline Pharmaceutical Co., Ltd. Carboprost was purchased from China Resources Zizhu Pharmaceutical Co., Ltd. Diclofenac was purchased from Renhe Pharmaceutical Co., Ltd. Fluoxetine was purchased from Sinochem Pharmaceutical Industry Co., Ltd. Phenylbutazone was purchased from Teyi Pharmaceutical Group Co., Ltd. Lupron was purchased from Shandong Luxi Pharmaceutical Co., Ltd. Vitamin A, vitamin B1, vitamin B5, vitamin D, and vitamin E were purchased from Huazhong Pharmaceutical Co., Ltd. Nickel foam was purchased from Hangzhou Dongying Co., Ltd. The ultrapure water used in the experiment was double distilled water.

## **Characterization apparatus**

The morphology of the materials was observed through scanning electron microscope (SEM, JSM-6700F), and the corresponding SEM images and energy dispersive spectrometer (EDS) were obtained. In addition, the microstructure of the material was analyzed using transmission electron microscope (TEM, JEOL JEM-2010, acceleration voltage of 200 kV) to obtain TEM images, high resolution transmission TEM images (HRTEM), element mapping and selected area electron diffraction image (SAED). For the identification of the crystal structure and chemical composition, X-ray diffraction (XRD, Rigaku D/Max-II B, using graphite monochromatic Cu Kα radiation, λ value is 0.15418 nm) and X-ray photoelectron spectroscopy (XPS, ESCALAB 250, using Mg Kα X-rays as the excitation source) were used. The measurement of zeta potential was performed by Zetasizer NanoZS of Malvern Instruments. The comparison surface area was testified by the dye-absorption tests with 0.5×2 cm^2^ of NF or NiMoS sample soaked in 10 mL Methyl Violet (MV) solution (4 mg L^-1^) and kept in the dark for 18 h. Ultraviolet–Visible Spectroscopy (UV-vis, UV-3100 UV VIS-NIR) was used for the optical testing. The electrochemical properties were measured on a CHI760e electrochemical workstation (Shanghai Chenhua Instrument Co., Ltd.) with a standard three-electrode system comprising a platinum wire as the auxiliary, a saturated calomel electrode (SCE) as the reference, and the modified GCE as the working electrode. The electrolytes used in the experiment were 0.1 M KCl and 5 mM [K_3_Fe(CN)_6_/K_2_Fe(CN)_6_] solutions. Electrochemical impedance spectroscopy (EIS) measurements were performed at open circuit potential with a frequency range from 0.01 Hz to 1000 kHz and an amplitude set to 5 mV. The obtained EIS data were analyzed using an equivalent circuit model. The typical equivalent circuit used to model the electrochemical interface includes: a solution resistance (*R_s_*), a double-layer capacitance (*C_dl_*), a charge transfer resistance (*R_ct_*), and a Warburg element (*Z_w_*). The equivalent circuit diagram is shown in Fig. 3a. The impedance data were fitted using ZView2 to extract the values of the circuit elements, particularly *R_ct_*. The fitting quality was evaluated using the chi-square (χ^2^) value and the fitting residuals to ensure an accurate representation of the experimental data. ^[1-3]^ The LSV (Linear Sweep Voltammetry) experiments were performed at a sweep rate of 5 mV s^-1^ with a potential window of 1.2 - 1.9 V (vs. RHE). And the potentials were calibrated concerning RHE, and the following equation was employed to convert the potentials from SCE to RHE.

$$E\left( vs. RHE \right) = E\left( vs. SCE \right) + 0.244 V + (0.0591 V)Ph$$

## **Electrochemiluminescence measurements**

The ECL measurements were carried out using a home-made ECL instrument, including a CHI760E electrochemical workstation (electrical energy supply), RFL-1 type ultraweak chemiluminescence detector (optical detector), and IFFS-A type multifunctional chemiluminescence detector (absolutely dark box). All ECL performances were employed with a classical three-electrode system: SCE as the reference electrode, Pt plate as the counter electrode, and GCE as the working electrode. Phosphate buffered saline (K_2_HPO_4_, KH_2_PO_4_, and KCl) containing tripropylamine (TPA) as the sacrificial coreactant were utilized as the electrolyte, and plots were collected over a potential ranging from 0 to +1.3 V with a scan rate of 120 mV s^-1^. Photomultiplier (PMT) was supplied at 500 V. When performing cyclic voltammetry (CV) experiments, we recorded the corresponding ECL signals.

## **Prepared sensor for Lidocaine detection**

Using Ru@NiMoS as the working electrode and 0.075 M PBS (pH 11) containing 100 μM NaNO_2_ as the supporting electrolyte, the assembled three-electrode system was used for the determination of lidocaine. The voltage of the PMT during detection was set to 800 V. Different concentrations of lidocaine (1 nM - 1 μM) were added to the supporting electrolyte and the corresponding ECL signals were recorded during CV scanning.


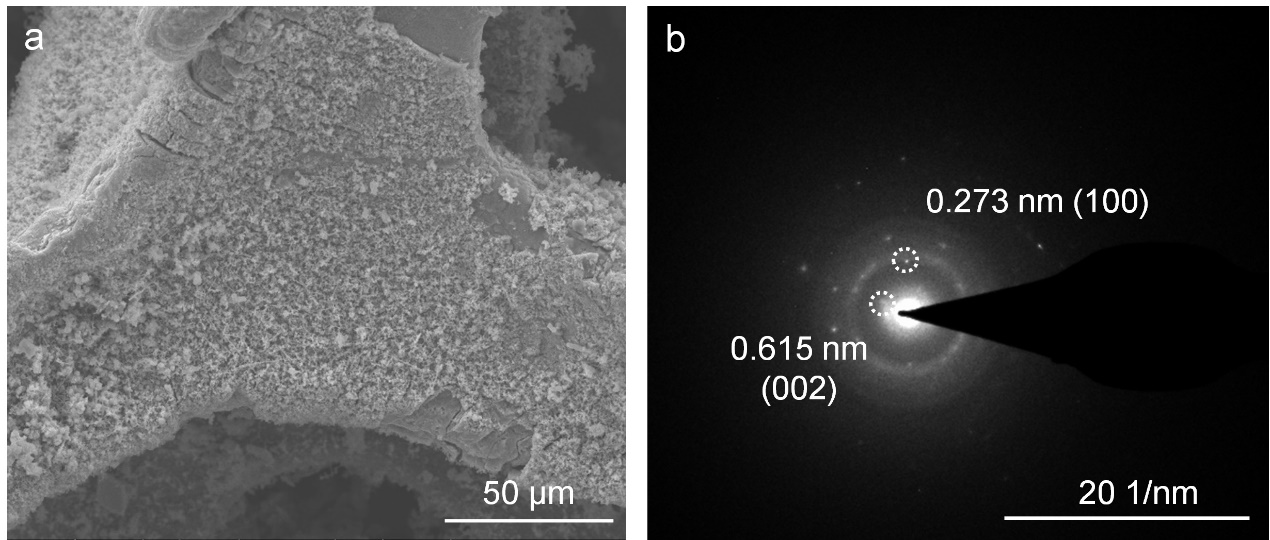


Fig. S1 (a) SEM, and (b) SEAD of NiMoS.

SEM image, as shown in Fig. S1a, revealed that NiMoS exhibited a three-dimensional faveolate structure. This structure was characterized by interconnected NiMoS sheets forming irregular pores, which significantly contributed to a substantial accessible surface area. Further, the SAED patterns, depicted in Fig. S1b, exhibited two distinct sets of concentric rings. These rings corresponded to the (002) and (100) planes of MoS_2_, indicating the crystalline nature of the material."


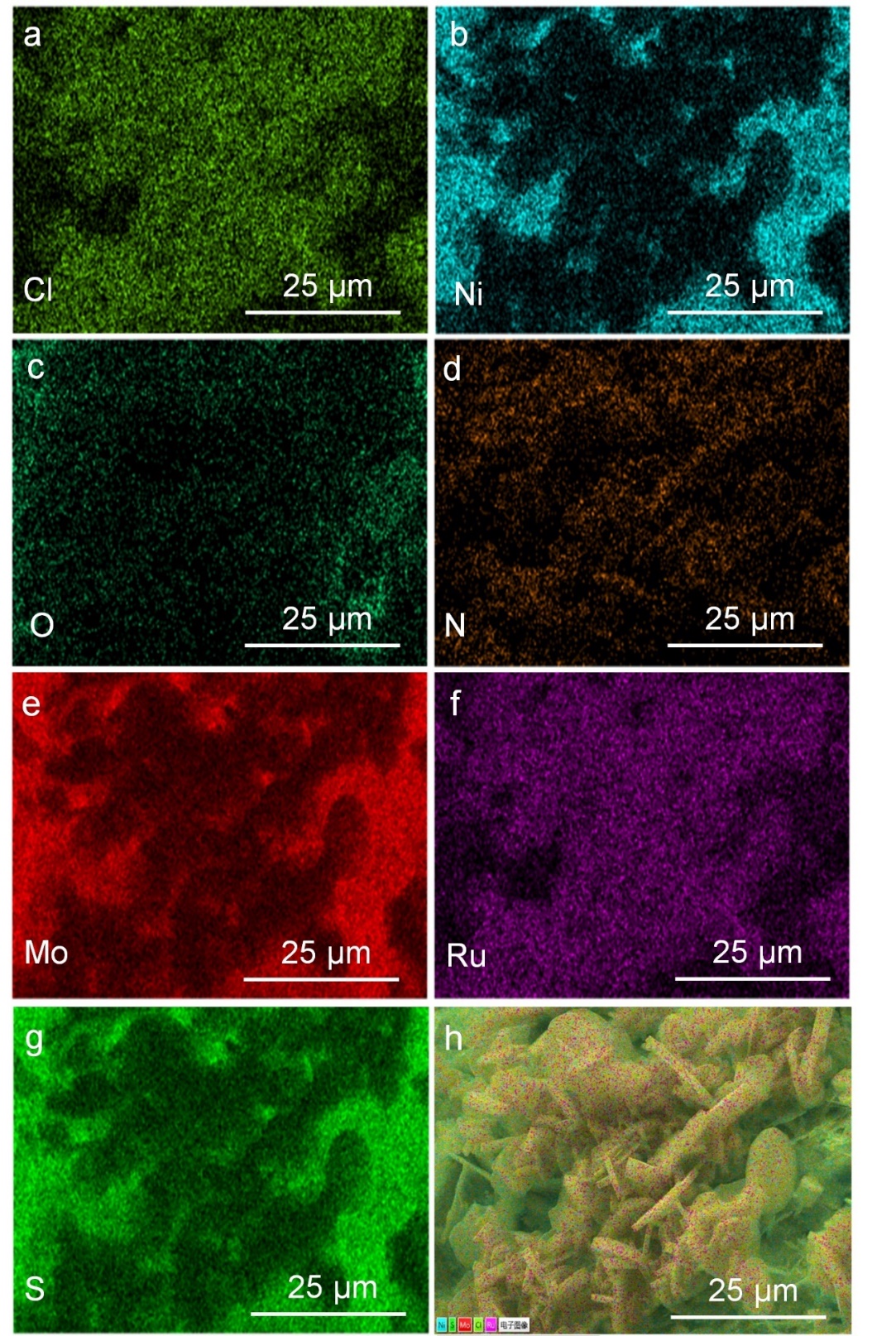


Fig. S2 EDS of (a) Cl, (b) Ni, (c) O, (d) N, (e) Mo, (f) Ru, (g) S and (h) mixed elemental mapping from Ru@NiMoS.

In Fig. S2, the element distributions of the as-prepared Ru@NiMoS composites were unraveled by EDS in Fig. S2a-g, in which the corresponding Ru, Cl, O, N, S, Ni and Mo elements were well-dispersed in the heterostructure, indicating NiMoS and Ru(bpy)_3_^2+^ were cross-linked to each other (Fig. S2h) via electrostatic interaction.


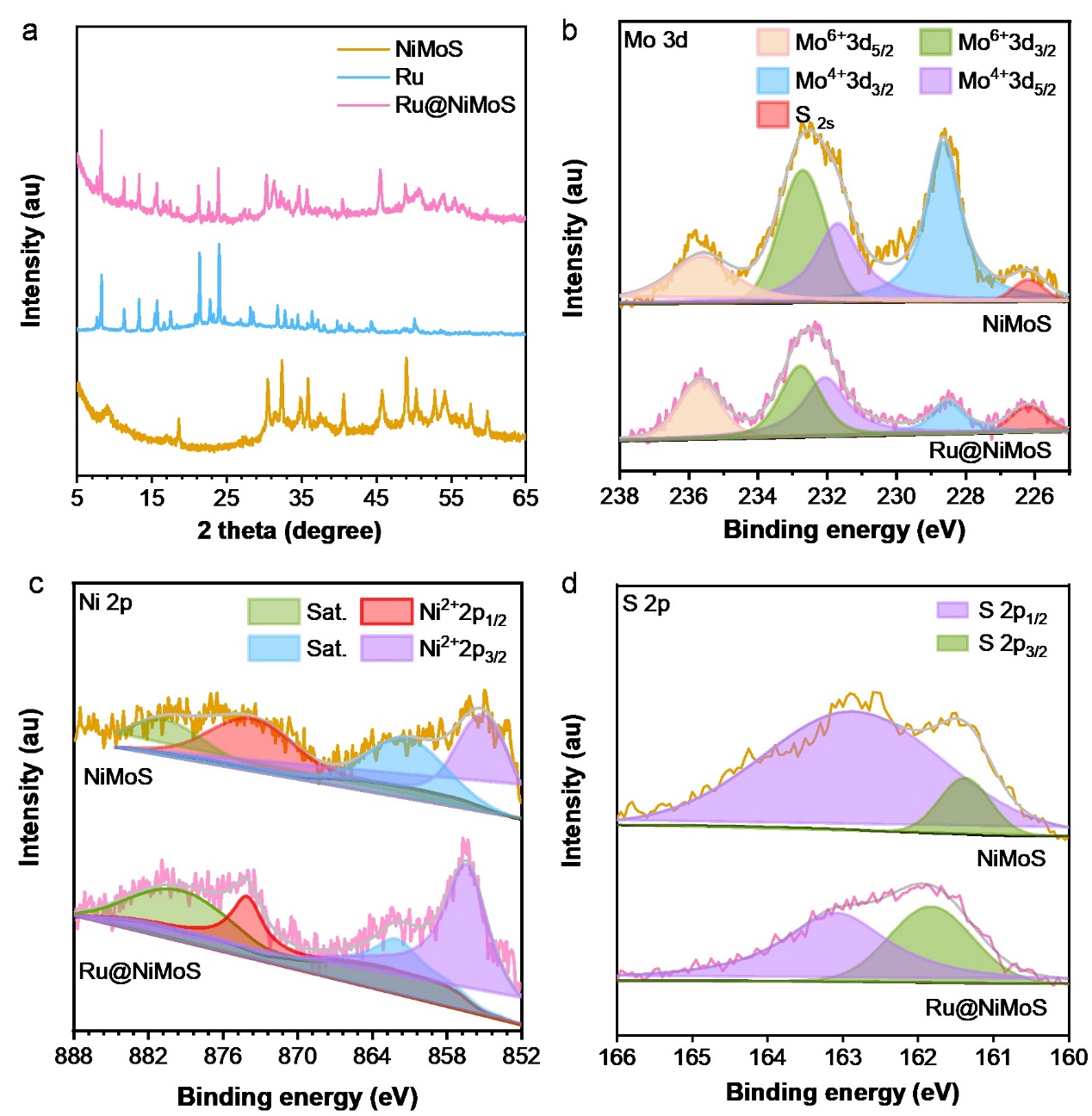


Fig. S3 (a) XRD pattern of NiMoS, Ru, and Ru@NiMoS. XPS high resolution spectra of (b) Mo 3d region, (c) Ni 2p region, and (d)S 2p region of NiMoS and Ru@NiMoS.

The composites’ structural characterizations were meticulously analyzed by XRD, as depicted in Fig S3a. The XRD pattern for NiMoS exhibited two distinct diffraction peaks attributed to the NiS (JCPDS 12-0041) and MoS_2_ (JCPDS 37-1492). The NiS crystalline planes stand out at 2-theta of 18.4°, 30.3°, 32.2°, 35.7°, 37.3°, 40.4°, 48.8°, 50.1°, 52.6°, 56.3°, 57.4°, and 59.7°, corresponding to (110), (101), (300), (021), (220), (211), (131), (410), (401), (321), (330), and (012) planes, respectively. Due to the relatively thin layer of MoS_2_ compared to the NiS background, the MoS_2_ XRD were less prominent, with only the 33.5°, 44.2°, and 55.9° peaks corresponding to (101), (006), (106) facets being discernible. Furthermore, the XRD analysis of Ru showed characteristic peaks of Ru(bpy)_3_^2+^ at 2θ values of 22.8°, 23.2°, 24.0°, 28.2°, 31.8, 32.8°, 33.7°, 41.4°, and 53.1°, aligning with previous reports. ^[4-7]^ The XRD pattern of Ru@NiMoS exhibited characteristic diffraction peaks of both NiMoS and Ru without the emergence of new planes, indicating that the attraction of Ru(bpy)_3_^2+^ exerted minimal influence on the crystal structure of NiMoS. Additionally, all three samples exhibited Ni (JCPDS 04-0850) diffraction peaks at 2θ values of 44.5°, 51.8°, and 76.4°, assigned to the crystal planes (111), (200), and (220), respectively, attributed to the elemental Ni substrate. XPS analysis provided deeper insight into the elemental composition and chemical states of the prepared samples. The Mo 3d spectrum, shown in Fig. S3b, featured a characteristic doublet for Mo^4+^ 3d^3/2^ and Mo^4+^ 3d^5/2^ at approximately 231.6 and 228.6 eV, respectively. Additionally, a pair of peaks at 235.7 eV and 232.7 eV were attributed to Mo^6+^ 3d_5/2_ and Mo^6+^ 3d_3/2_, indicating the ease of MoS_2_ oxidation. A subordinate peak at 226.4 eV, corresponding to the S 2s peak, was also observed. ^[8-11]^ Fig. S3c presented the Ni 2p XPS core spectra, showing two principal peaks at 855.7 and 873.4 eV, corresponding to Ni 2p_3/2_ and Ni 2p_1/2_ of Ni^2+^, along with two spin-orbit satellite peaks (labeled "Sat.") at binding energies of 861.3 eV and 879.6 eV. ^[12]^ In Fig. S3d, the S 2p spectrum displayed two peaks at 161.6 and 163.1 eV, consistent with the orbitals of divalent sulfide ions (S^2-^ 2p_3/2_ and S^2-^ 2p_1/2_) ^[13]^ Notably, compared to NiMoS, the S 2p nuclear energy levels in Ru@NiMoS shifted to the higher energy side by 400 meV, suggesting the formation of Ru-S ionic bonds. ^[14]^ Collectively, these findings indicated that the chemical structure of Ru(bpy)_3_^2+^ remained unchanged in the Ru@NiMoS sample, and that Ru(bpy)_3_^2+^ was successfully anchored on the surface of the NiMoS structure.


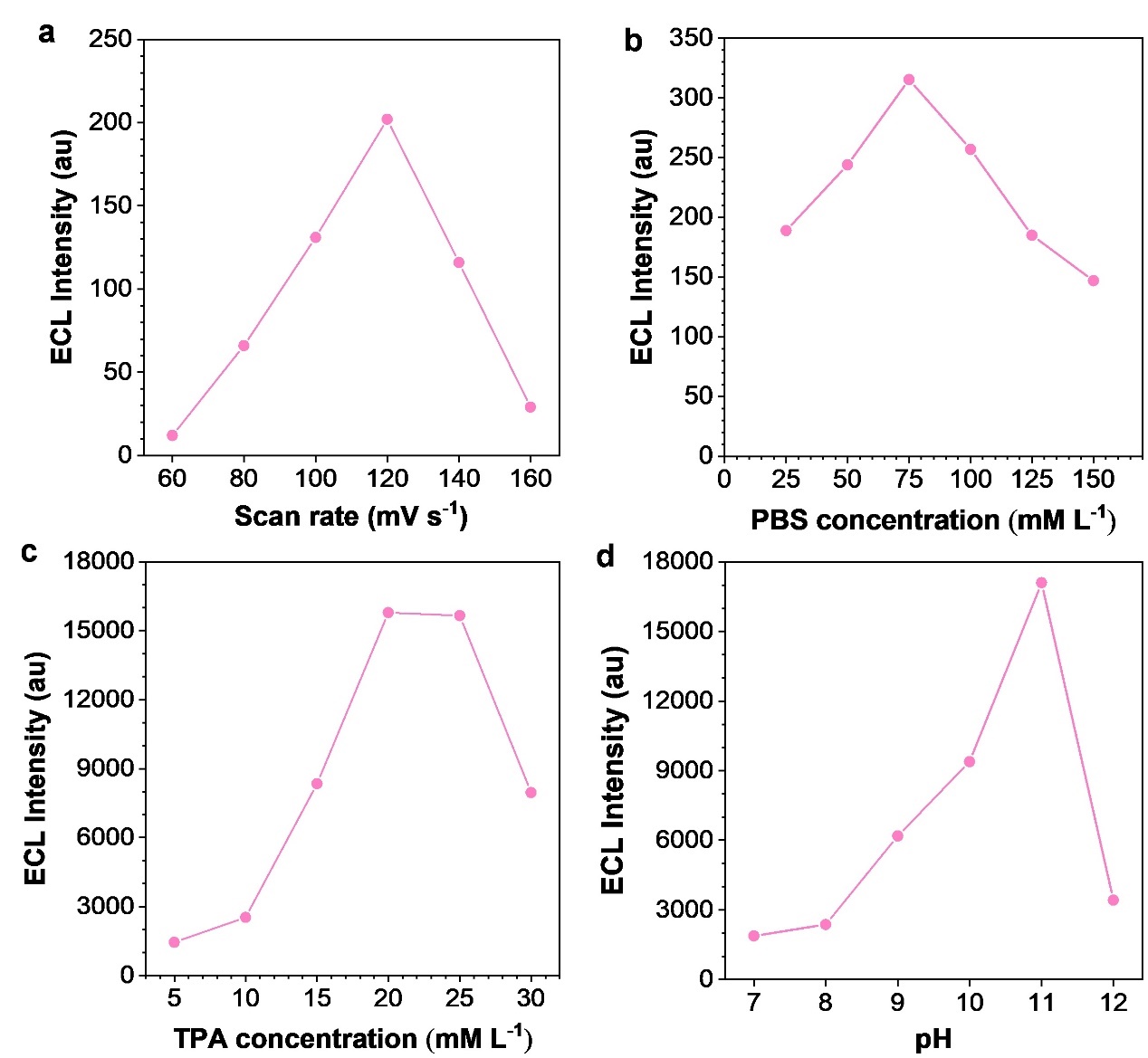


Fig. S4 Optimization of (a) scan rate, (b) PBS concentration, (c) TPA concentration, and (d) pH of the electrolyte for Ru@NiMoS.

The active ECL materials are significantly affected by the ionic strength, coreactant content and pH value of the supporting electrolyte. Additionally, the electrochemical reaction rate and the generation of the excited state are contingent upon the scan rate. As illustrated in Fig. S4a, an increase in scan rate initially led to a rise in the ECL intensity, which peaked at a scan rate of 120 mV s^-1^, before subsequently diminishing. Further exploration, as shown in Fig. S4b, indicated that 0.075M PBS provides an optimal buffer environment for Ru@NiMoS, surpassing the luminescence intensity achieved with the conventional concentration of 0.1 M by 1.3 times. This suggested that the diffusion resistance of active ions was minimized at this specific concentration. The incorporation of the coreactant TPA significantly enhanced the ECL emission, as evidenced in Fig. S4c. This enhancement was particularly notable at a TPA concentration of 20 mM, where the ECL intensity reached 1589 au, underscoring the inherent coreactant ECL properties of the Ru@NiMoS luminescent aggregate. Fig. S4d studied the effect of the pH value of the electrolyte on the ECL intensity of Ru@NiMoS. The findings revealed that maximal emission was attained under the alkaline condition, specifically at a pH of 11. In summary, the optimal conditions for achieving maximum ECL intensity in Ru@NiMoS were established as using 0.075 M PBS at a pH of 11, adding 20 mM TPA, and employing a scan rate of 120 mV s^-1^.


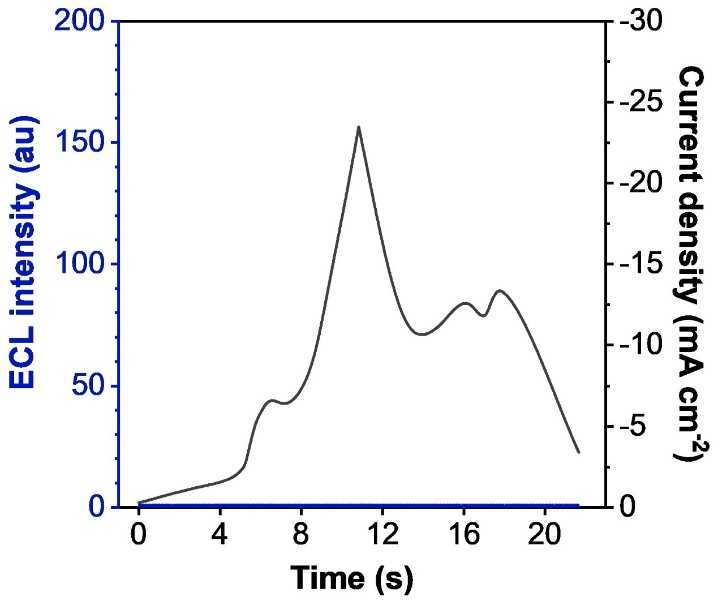


Fig. S5 Synchronized ECL and CV curves of NiMoS (PMT: 500 V, electrolyte: 0.075 M PBS (pH 11) with 20 mM TPA, potential range: 0 - 1.3 V, scan rate: 120 mV s^-1^).

To investigate the influence of the NiMoS substrate on ECL, the luminescent property of NiMoS in isolation was examined, as detailed in Fig. S5. The findings revealed that NiMoS did not exhibit any luminescent response at its current peak position. This observation conclusively affirmed the inherent non-luminescent nature of NiMoS.


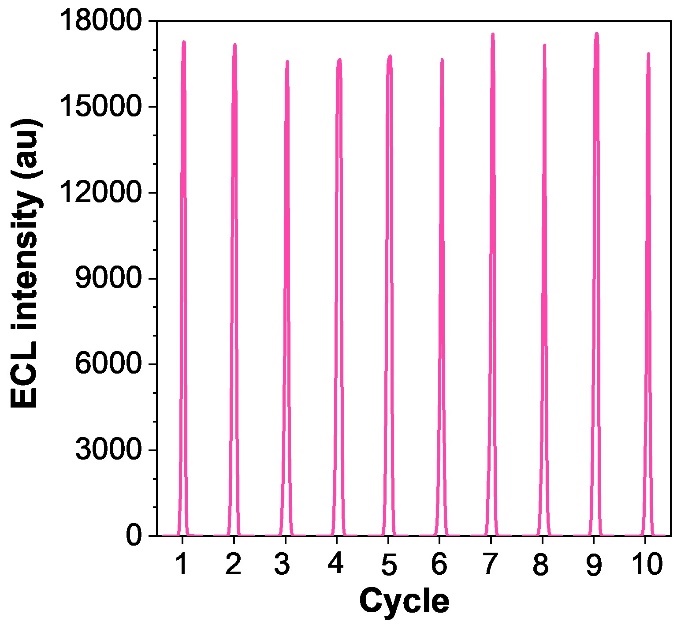


Fig. S6 Repeated ECL tests of Ru@NiMoS (PMT: 500 V, electrolyte: 0.075 M PBS (pH 11) with 20 mM TPA, potential range: 0 - 1.3 V, scan rate: 120 mV s^-1^).

The repeatability of Ru@NiMoS is critical in demonstrating its reliability. As illustrated in Fig. S6, remarkable consistency was performed across 10 separate scans, evidenced by a notably low RSD of only 0.53%.


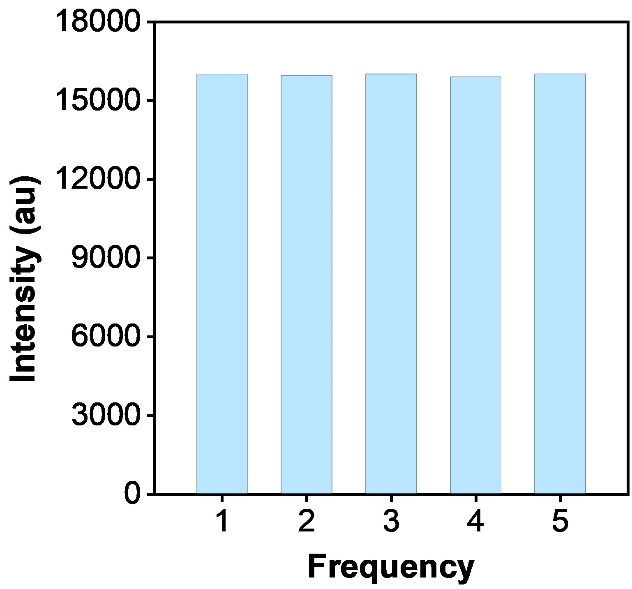


Fig. S7 Reproducibility of five Co₃O₄@Ru-modified electrodes tested with 1 μM lidocaine (PMT: 800 V, electrolyte: 0.075 M PBS (pH 11) with 100 μM NO₂⁻, potential range: 0 - 1.3 V, scan rate: 120 mV s⁻¹).

Fig. S7 demonstrates the reproducibility of the Ru@NiMoS sensor for lidocaine detection, showing exceptional consistency across 5 attempts, with an impressive low RSD of 0.31%

Table S1 Atomic percent derived from XPS data for NiMoS and Ru@NiMoS.

| **Elements** | **NiMoS** | **Ru@NiMoS** |
| --- | --- | --- |
| Mo : S | 10.2% | 23.7% |
| Ni : S | 18.3% | 47.8% |

To substantiate the formation of coordination fixation, a detailed analysis of the surface composition and stoichiometry of the thin films was conducted. This involved calculating the precise percentages of elements from the area under XPS peaks, with the results presented in Table S1. NiMoS showed the Mo:S ratio of ~10.2%, while Ru@NiMoS showed the content of ~23.7%, in which the raising ratio related to the adsorption and catalysis of O_2_. ^[15]^ Regarding the Ni:S ratio, values of 18.3% for NiMoS and 47.8% for Ru@NiMoS were calculated. The disparity in sulfur content between the two samples was noteworthy, with Ru@NiMoS exhibiting a significantly lower number of surface sulfur atoms compared to NiMoS. This difference supported to the hypothesis that sulfur atoms play a pivotal role in anchoring the ruthenium element within the structure. ^[16]^

Table S2 Summary of data for lidocaine detection in real sample based on Ru@NiMoS sensor.

| **Samples** | **Added** | **Found** | **Recovery** | **RSD** |
| --- | --- | --- | --- | --- |
|  | 0 | Not Detected |  |  |
| Human-serum sample | 10 µM | 9.26 µM,  10.34 µM,  9.87 µM | 92.6%  103.4%  98.7% | 5.4% |
|  | 1 µM | 0.95 µM,  1.09 µM,  0.91 µM, | 95%  109%  91% | 9.4% |
|  | 100 nM | 106.73 nM,  101.86 nM,  97.23 nM | 106.73%  101.86%  97.23% | 4.7% |

In this study, we investigated the feasibility of a Ru@NiMoS-based sensor for analyzing lidocaine in human serum samples. As detailed in Table S2, we conducted a comprehensive analysis of serum samples with varying concentrations of lidocaine (10 μM, 1 μM, and 100 nM). The proposed E-MRu-based sensor demonstrated reasonable recovery rates across all concentration levels, ranging from 92.6% to 103.4%, 91% to 109%, and 97.23% to 106.73%, respectively. Additionally, the RSD for all samples were below 10%. These findings suggest that our Ru@NiMoS-based sensor offers significant accuracy and reliability for the real-time monitoring of lidocaine.

**Reference**

1. A. J. Bard, L. R. Faulkner, H. S. [White](https://scholar.google.com/citations?user=lNxH8sQAAAAJ&hl=zh-CN&oi=sra), *Electrochemical Methods: Fundamentals and Applications*. **2022**.
2. J. R. Macdonald, W. B. Johnson, *Impedance spectroscopy: theory, experiment, and applications*, **2018**, 1-20.
3. V. F. Lvovich, *Impedance Spectroscopy: Applications to Electrochemical and Dielectric Phenomena.* **2012**.
4. J. Marques, L. Anjo, M. P. M. Marques, T. M. Santos, F. A. A. Paz, S. S. Braga, *Journal of Organomet. Chem.* **2008**, 693, 3021-3028.
5. J. Zhou, R. Zong, J. Ye, *J. Lumin.* **2007**, 122, 218-220.
6. S. Chinnathambi, M. Ammam, *J. Power Sources.* **2015**, 284, 524-535.
7. T. A. Ho, Y. B. Cho, Y. S. Kim, *J. Nanosci. Nanotechno.* **2016**, 16, 4534-4538.
8. L. F. Yang, L. Zhang, G. C. Xu, X. Ma, W. W. Wang, H. J. Song, D. Z. Jia, *ACS Sustain. Chem. Eng.* **2018**, 6, 12961–12968.
9. M. X. Shang, C. C. Du, H. Huang, J. X. Mao, P. Liu, W. B. Song, *J. Colloid Interface Sci.* **2018**, 532, 24–31.
10. D. Siddhartha, T. Abhishek, H. Shamima, *Mater. Today: Proc.* **2021**, 46, 6127-6131.
11. H. Y. Nan, Z. L. Wang, W. H. Wang, Z. Liang, Y. Lu, Q. Chen, D. W. He, P. H. Tan, F. Miao, X. R. Wang, J. L. Wang, Z. H. Ni, *ACS Nano* **2014**, 8, 5738-5745.
12. X. B. Xu, W. Zhong, X. Zhang, J. Dou, Z. G. Xiong, Y. Sun, T. T. Wang, Y. W. Du, *J. Colloid Interface Sci.* **2019**, 543, 147–155.
13. M. A. Bissett, I. A. Kinloch, R. A., Dryfe, *ACS Appl. Mater. Interfaces* **2015**, 7, 17388–17398.
14. S. Baik, Y. Koo, W. Choi, *Curr. Appl. Phys.* **2022**, 42, 38-42.
15. K. Saha, S. Gayen, U. Kaur, T. Roisnel, S. Ghosh, *Dalton Trans.* **2021**, 50, 12990-13001.
16. S. Dam, A. Thakur, S. Hussain, *Mat. Sci. Semicon. Proc.* **2021**, 136, 106162.
